# TARGETING AND APPROACHING LEGISLATURES TO SUPPORT FAMILY CAREGIVING: LESSONS LEARNED

**DOI:** 10.1093/geroni/igad104.0826

**Published:** 2023-12-21

**Authors:** Beth Fields

**Affiliations:** University of Wisconsin-Madison, Madison, Wisconsin, United States

## Abstract

The Caregiver Advise, Record, and Enable (CARE) Act has passed in 45 states and requires hospitals to ask patients if they have a family caregiver, notify that family caregiver about the patients upcoming discharge, and educate family caregivers how to provide the care the patient will need after discharge. Early research demonstrates that this Act has favorably influenced hospital and patient outcomes such as quality indicators and family caregiver confidence. Despite this research and widespread support, this Act has yet to be passed in Wisconsin. This presentation will report on lessons learned from a systematic method for targeting and approaching Wisconsin state legislatures within the Assembly Committee on Health, Aging, and Long-term Care. Interviews, fieldnotes, in-person visits, and telephone and email correspondences with legislatures were conducted to learn their perspectives on advancing family caregiving policy. Lessons learned from this systematic method for targeting and approaching legislatures included the timing of interactions matter, the use of concise language maximizes understanding, the mode of delivery impacts response rate, and the transformation of family caregiving research to infographics increases uptake. Findings from this work can be used to elevate family caregiving support by bridging the evidence to policy gap.

